# Influenza D Virus in Cattle, Ireland

**DOI:** 10.3201/eid2402.170759

**Published:** 2018-02

**Authors:** Orla Flynn, Clare Gallagher, Jean Mooney, Claire Irvine, Mariette Ducatez, Ben Hause, Guy McGrath, Eoin Ryan

**Affiliations:** Department of Agriculture, Food, and Marine Laboratory Services, Celbridge, Ireland (O. Flynn, C. Gallagher, J. Mooney, C. Irvine, E. Ryan);; École Nationale Vétérinaire de Toulouse, Toulouse, France (M. Ducatez);; Kansas State University College of Veterinary Medicine, Manhattan, Kansas, USA (B. Hause);; University College Dublin, Dublin, Ireland (G. McGrath)

**Keywords:** influenza D virus, viruses, influenza, cattle, respiratory infections, Ireland

## Abstract

We detected influenza D virus in 18 nasal swab samples from cattle in Ireland that were clinically diagnosed with respiratory disease. Specimens were obtained from archived samples received for routine diagnosis during 2014–2016. Sequencing showed that viruses from Ireland clustered with virus sequences obtained in Europe within the D/swine/OK/1334/2011 clade.

Influenza D virus is a recently characterized addition to the family *Orthomyxoviridae*. This virus was originally detected in pigs in the United States (*1*); however, cattle are now believed to be the main reservoir species (*2*). Evidence suggests that this virus plays a role in bovine respiratory disease, although experimentally, it caused only mild disease by itself (*3*). Influenza D virus has been found to be associated with respiratory disease in feedlot cattle (*4*).

The zoonotic potential of influenza D virus remains unclear; this virus can replicate in ferrets (a model for human influenza infection), and a seroprevalance of 91% was found in persons working closely with cattle (*5*). However, a study of 3,300 human respiratory samples from Scotland did not detect any influenza D virus–positive samples (*6*). This virus has been detected in bovine samples in several other countries, including France (*7*), Italy (*8*), Japan, and China (*9*).

Cattle are a major part of the economy in Ireland, where there are ≈7 million (*10*). To determine whether influenza D virus was present in cattle in Ireland and to investigate epidemiologic factors that might be related to this virus, we conducted a cross-sectional study by using 320 nasal swab specimens from cattle with respiratory disease that were submitted to the Central Veterinary Research Laboratory (Celbridge, Ireland) for routine bovine viral pathogen testing during 2014–2016.

We tested swab specimens by using real-time PCR for influenza D virus as described (*1*). We selected samples with a cycle threshold (C_t_) <25 for further molecular characterization by using 3 primer sets (*7*) that are specific for the 7 virus gene segments. We performed cDNA synthesis by using qScript cDNA SuperMix (Quantabio, Beverly, MA, USA) and PCR amplification by using AccuStart II PCR ToughMix (Quantabio).We processed PCR products by using Illustra ExoProStar 1-Step (GE Healthcare, Little Chalfont, UK) according to the manufacturer’s instructions before Sanger sequencing. We analyzed sequence data by using DNASTAR Lasergene 12 SeqMan Pro (DNASTAR, Madison, WI, USA) and performed sequence alignment by using ClustalW in MEGA 5.01 (http://www.megasoftware.net/). We constructed phylogenetic trees by using the maximum-likelihood method in MEGA 5.01.

Herd information for 2015 was available for 84 herds of origin of these nasal swab specimens. Data were obtained from the Animal Identification and Movement System database of the Department of Agriculture, Food, and Marine Laboratory Sciences of Ireland (https://www.agriculture.gov.ie/animalhealthwelfare/animalidentificationmovement/cattle/irishbovineanimalidentificationsystem-overview/). We performed univariate statistical analysis by using Stata/SE14.1 (StataCorp LLC, College Station, TX, USA). Herd factors investigated for a possible association with influenza D virus herd status were herd size, numbers of stillbirths, dairy cows in herd, beef cows in herd, inward movements from markets, inward movements to farm, and carcasses moved to knackeries.

A total of 18/320 samples were positive for influenza D virus by PCR. Of the 18 positive samples, 13 were also positive by PCR for 1 or 2 other viral pathogens (bovine herpesvirus 1, parainfluenza 3 virus, bovine coronavirus, bovine respiratory syncytial virus, bovine viral diarrhea virus). Seven of the influenza D virus–positive specimens were from calves, 2 from weanlings, and 1 from a cow; other specimens were not described by animal age. Nine of the influenza D virus–positive samples had a C_t_ <25 and were selected for sequencing. We obtained partial sequences for 5 samples and deposited the sequences in GenBank (accession nos. KY992090–KY992103).

Phylogenetic analysis (Technical Appendix Figure 1) showed that the 5 influenza D virus isolates from Ireland clustered with viruses from Europe in the D/swine/OK/1334/2011 clade. We also determined the distribution of positive and negative samples at county level in Ireland (Technical Appendix Figure 2).

Herd information was available for 10 of the influenza D virus–positive herds and for 74 comparison herds (for which the nasal swab specimens were negative for influenza D virus but which had clinical respiratory disease outbreaks). We found no associations between herd characteristics and influenza D virus status; this finding was determined by evaluating mean values with 95% CIs for infected herds and noninfected herds.

This study confirms the emergence of influenza D virus in Ireland. Presence of the virus in nasal swab specimens submitted from routine respiratory disease cases supports the hypothesis that this virus plays a role in the bovine respiratory disease complex. Analysis of herds for infected cattle did not show any epidemiologic differences between influenza D virus infection and infection with other common respiratory viral pathogens. This finding is consistent with the hypothesis that influenza D virus might have limited effect by itself but can potentiate effects of other respiratory pathogens in causing respiratory disease (*3*,*4*).

Detection of 2 virus lineages in Ireland clustering with viruses isolated in Europe within the D/swine/OK/1334/2011 clade raises the issue of how influenza D virus might spread internationally. Surveillance efforts could be targeted for data on trade of live cattle, which is extensive within Europe. Further research is planned to investigate the seroprevalence of influenza D virus in cattle in Ireland and to determine the effect of this virus in a cattle farming context in this country.

Technical Appendix Additional information on influenza D virus in cattle, Ireland.

