# Supplementary material for: Influenza D Virus in Cattle, Ireland
Source: Emerg Infect Dis. 2018 Feb;24(2):389–91. doi: 10.3201/eid2402.170759 (PMC5782902; doi:10.3201/eid2402.170759)
Supplement: Technical Appendix — Additional information on influenza D virus in cattle, Ireland. [file 17-0759-Techapp-s1.pdf]

Article DOI: https://doi.org/10.3201/eid2402.170759

Influenza D Virus in Cattle, Ireland

Technical Appendix

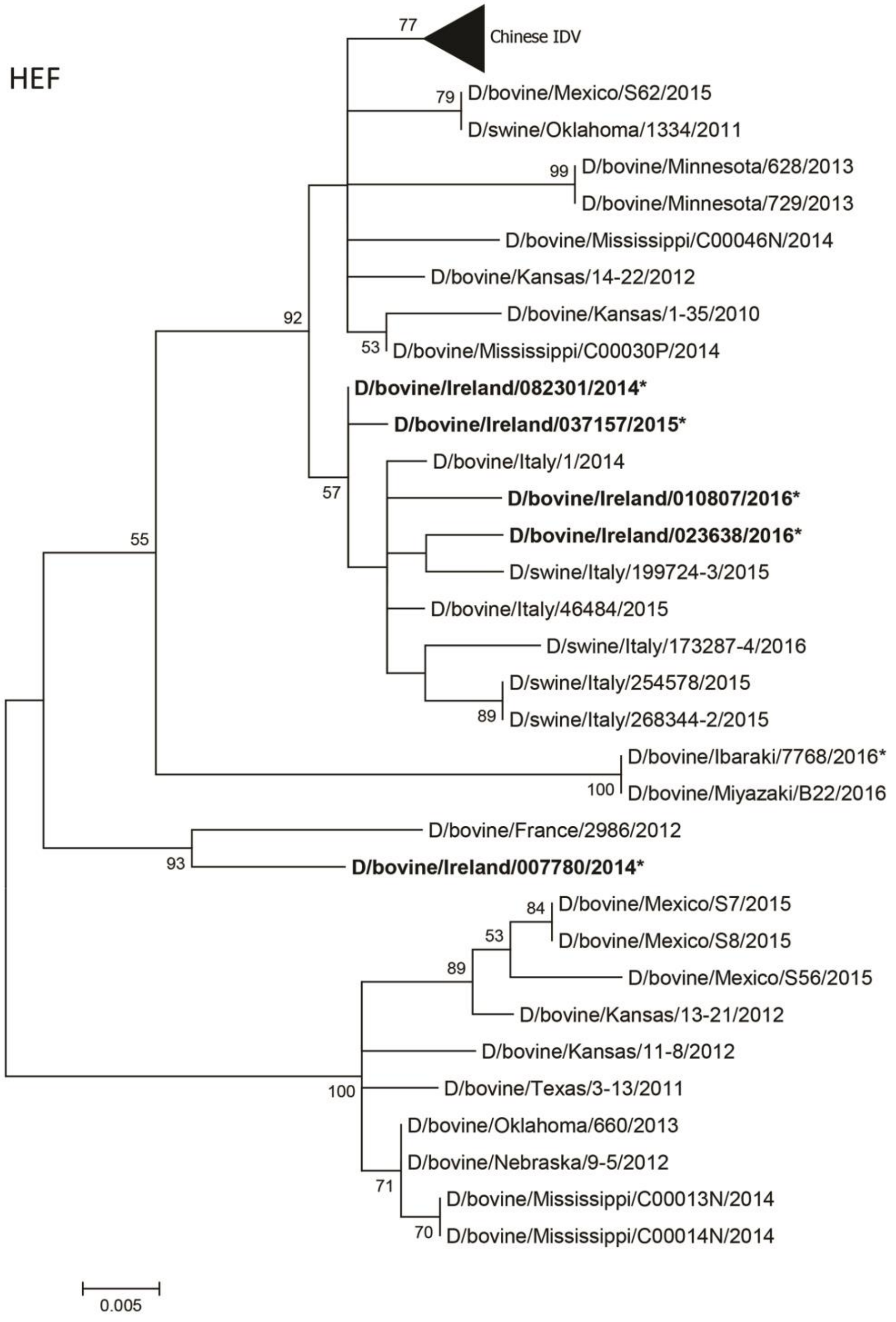

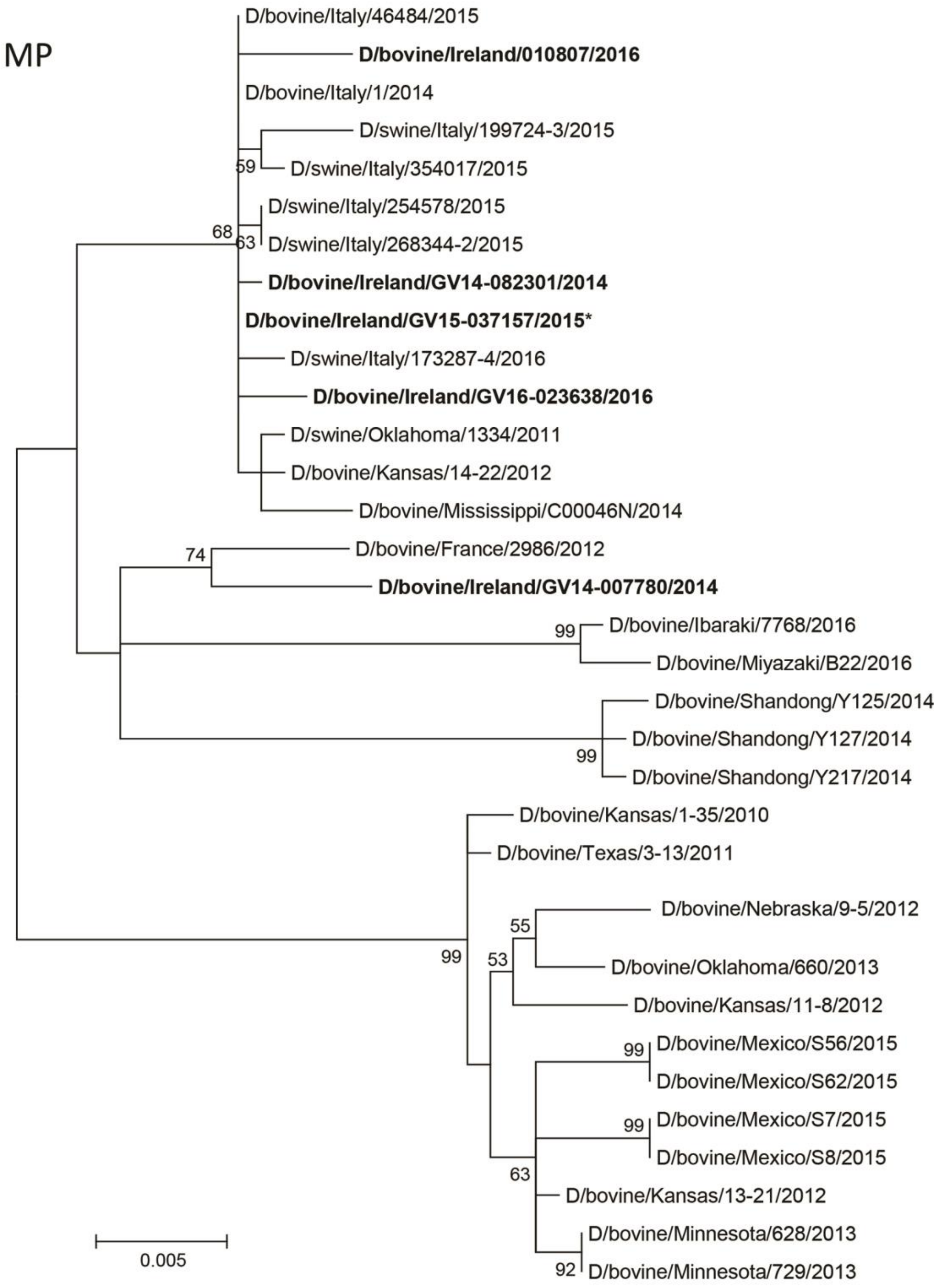
MP
D/bovine/Italy/46484/2015
D/bovine/Ireland/010807/2016
D/bovine/Italy/1/2014
D/swine/Italy/199724-3/2015
59
D/swine/Italy/354017/2015
D/swine/Italy/254578/2015
68
63
D/swine/Italy/268344-2/2015
D/bovine/Ireland/GV14-082301/2014
D/bovine/Ireland/GV15-037157/2015*
D/swine/Italy/173287-4/2016
D/bovine/Ireland/GV16-023638/2016
D/swine/Oklahoma/1334/2011
D/bovine/Kansas/14-22/2012
D/bovine/Mississippi/C00046N/2014
74
D/bovine/France/2986/2012
D/bovine/Ireland/GV14-007780/2014
99
D/bovine/Ibaraki/7768/2016
D/bovine/Miyazaki/B22/2016
D/bovine/Shandong/Y125/2014
D/bovine/Shandong/Y127/2014
99
D/bovine/Shandong/Y217/2014
D/bovine/Kansas/1-35/2010
D/bovine/Texas/3-13/2011
D/bovine/Nebraska/9-5/2012
55
99
53
D/bovine/Oklahoma/660/2013
D/bovine/Kansas/11-8/2012
99
D/bovine/Mexico/S56/2015
D/bovine/Mexico/S62/2015
99
D/bovine/Mexico/S7/2015
D/bovine/Mexico/S8/2015
63
D/bovine/Kansas/13-21/2012
D/bovine/Minnesota/628/2013
92
D/bovine/Minnesota/729/2013
0.005

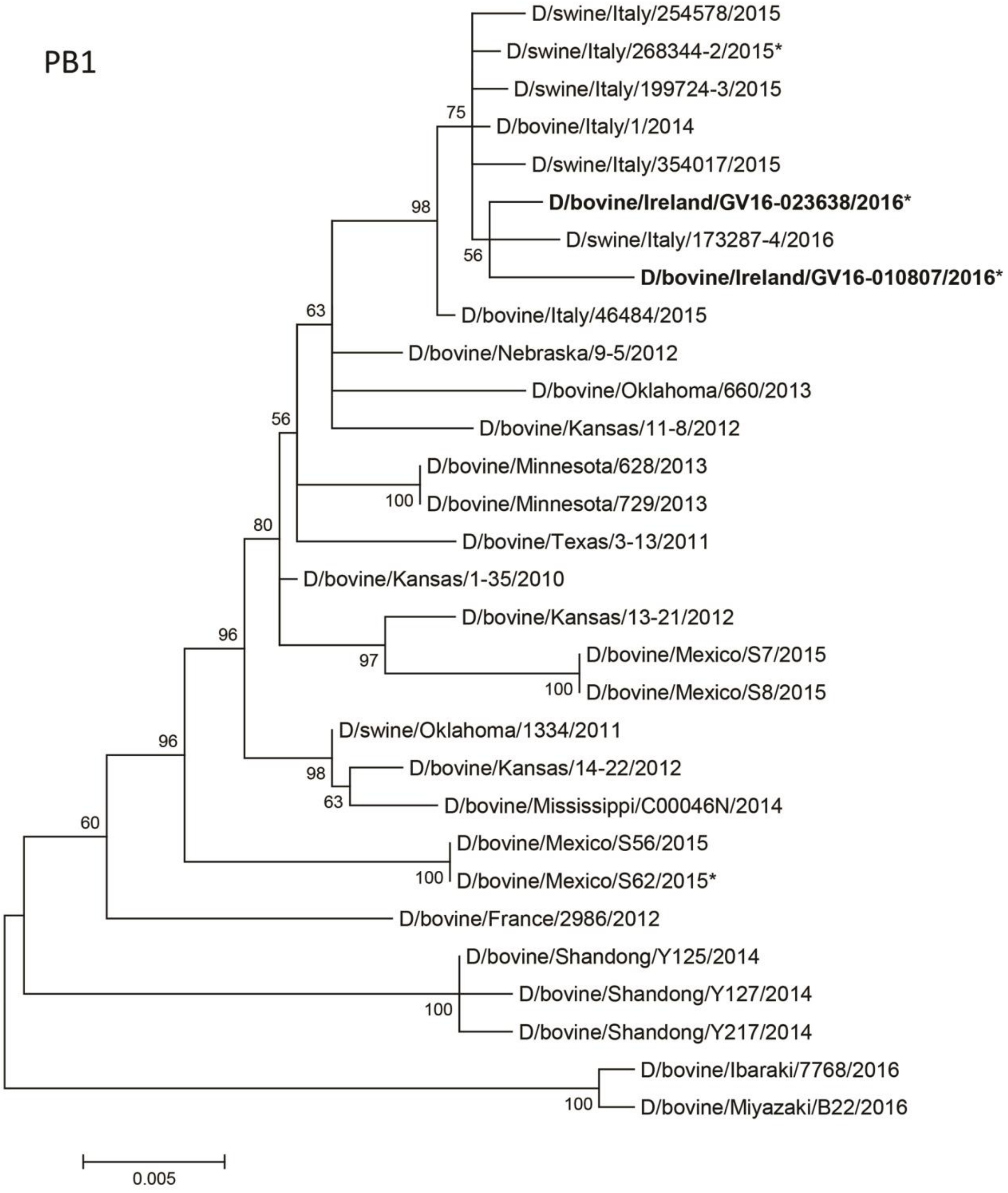

PB1
D/swine/Italy/254578/2015
D/swine/Italy/268344-2/2015*
D/swine/Italy/199724-3/2015
75
D/bovine/Italy/1/2014
D/swine/Italy/354017/2015
98
D/bovine/Ireland/GV16-023638/2016*
D/swine/Italy/173287-4/2016
56
D/bovine/Ireland/GV16-010807/2016*
63
D/bovine/Italy/46484/2015
D/bovine/Nebraska/9-5/2012
D/bovine/Oklahoma/660/2013
56
D/bovine/Kansas/11-8/2012
D/bovine/Minnesota/628/2013
100
D/bovine/Minnesota/729/2013
80
D/bovine/Texas/3-13/2011
D/bovine/Kansas/1-35/2010
D/bovine/Kansas/13-21/2012
96
97
D/bovine/Mexico/S7/2015
100
D/bovine/Mexico/S8/2015
D/swine/Oklahoma/1334/2011
96
98
D/bovine/Kansas/14-22/2012
63
D/bovine/Mississippi/C00046N/2014
60
D/bovine/Mexico/S56/2015
100
D/bovine/Mexico/S62/2015*
D/bovine/France/2986/2012
D/bovine/Shandong/Y125/2014
D/bovine/Shandong/Y127/2014
100
D/bovine/Shandong/Y217/2014
D/bovine/Ibaraki/7768/2016
100
D/bovine/Miyazaki/B22/2016
0.005

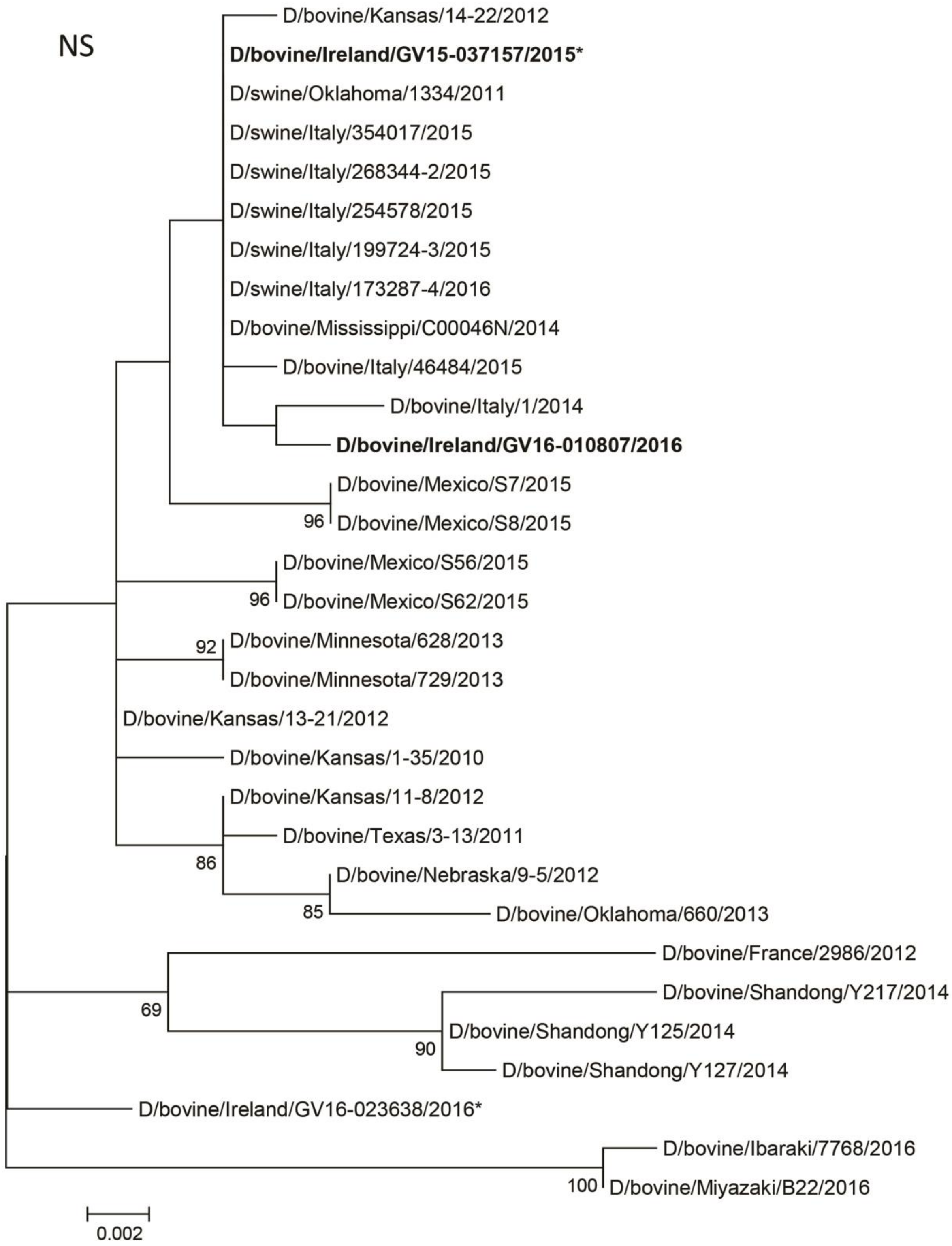
NS
D/bovine/Kansas/14-22/2012
D/bovine/Ireland/GV15-037157/2015*
D/swine/Oklahoma/1334/2011
D/swine/Italy/354017/2015
D/swine/Italy/268344-2/2015
D/swine/Italy/254578/2015
D/swine/Italy/199724-3/2015
D/swine/Italy/173287-4/2016
D/bovine/Mississippi/C00046N/2014
D/bovine/Italy/46484/2015
D/bovine/Italy/1/2014
D/bovine/Ireland/GV16-010807/2016
D/bovine/Mexico/S7/2015
96
D/bovine/Mexico/S8/2015
D/bovine/Mexico/S56/2015
96
D/bovine/Mexico/S62/2015
92
D/bovine/Minnesota/628/2013
D/bovine/Minnesota/729/2013
D/bovine/Kansas/13-21/2012
D/bovine/Kansas/1-35/2010
D/bovine/Kansas/11-8/2012
D/bovine/Texas/3-13/2011
86
D/bovine/Nebraska/9-5/2012
85
D/bovine/Oklahoma/660/2013
D/bovine/France/2986/2012
D/bovine/Shandong/Y217/2014
69
D/bovine/Shandong/Y125/2014
90
D/bovine/Shandong/Y127/2014
D/bovine/Ireland/GV16-023638/2016*
D/bovine/Ibaraki/7768/2016
100
D/bovine/Miyazaki/B22/2016
0.002

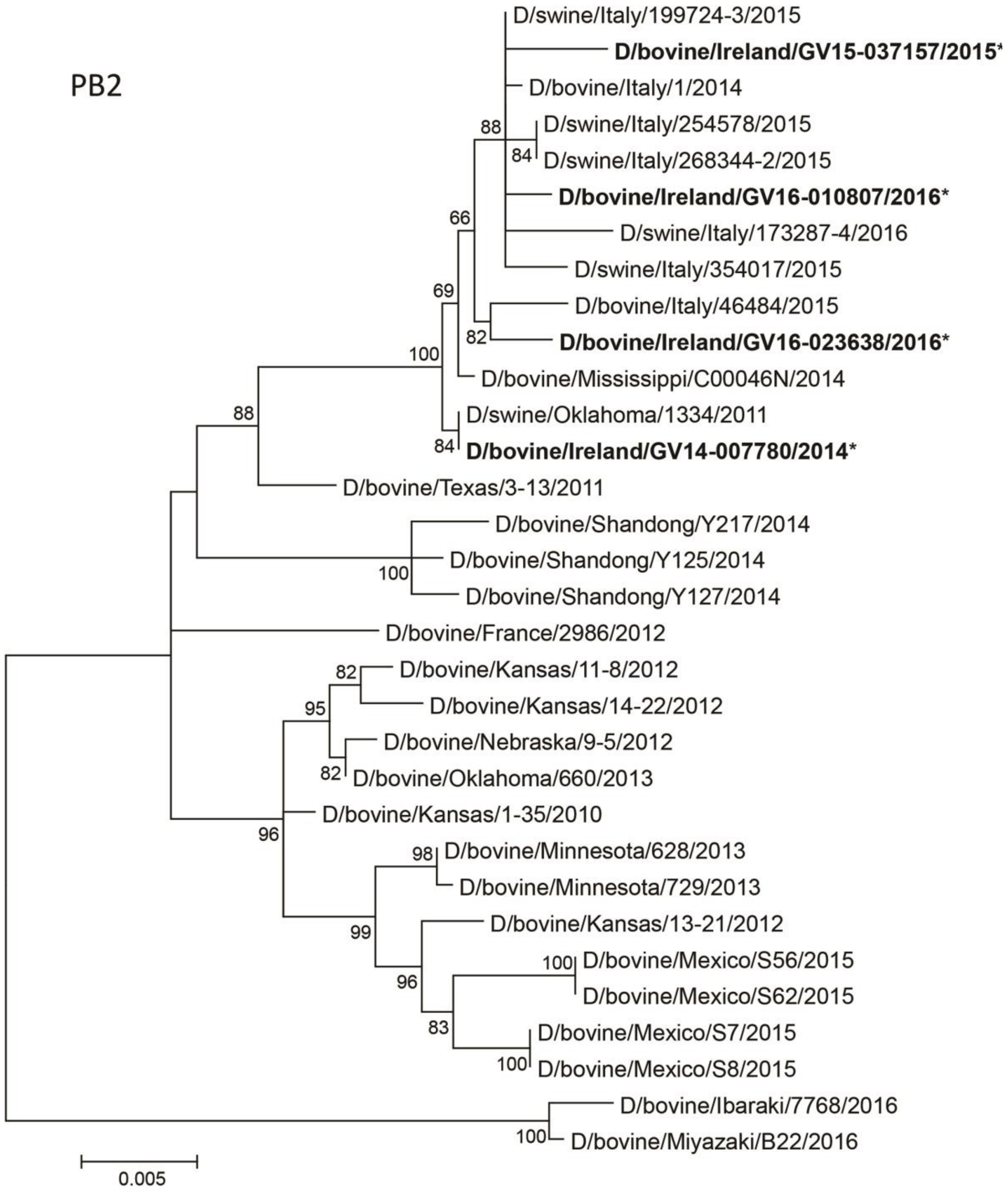

Technical Appendix Figure 1. Phylogenetic trees for 7 genomic segments of influenza D viruses obtained from cattle in Ireland and comparison with virus sequences obtained from GenBank. *Indicates incomplete sequences (only bootstraps >50 are shown). Bold indicates strains isolated in this study. Scale bars indicate nucleotide substitutions per site. PB1, polymerase basic 1; PB2, polymerase basic 2; P3, polymerase 3; HEF, hemagglutinin esterase fusion; NP, nucleoprotein; MP, matrix protein; NS, nonstructural.

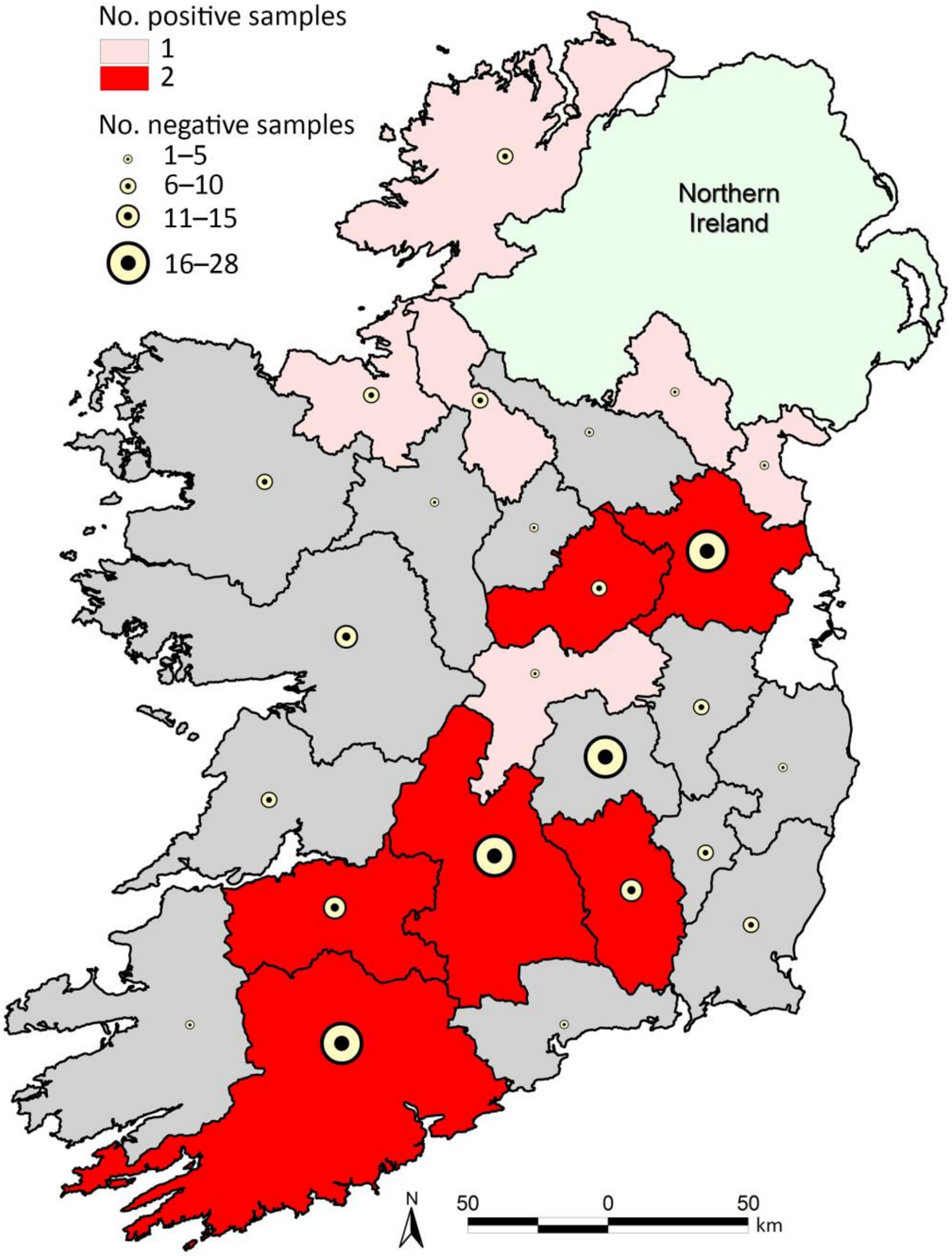

Technical Appendix Figure 2. Geographic distribution of positive and negative samples for influenza D virus, by county, in Ireland. Pink indicates 1 positive detected; red indicates 2 positive samples detected; gray indicates no positive samples detected. Circles indicate number of negative samples per county.
